# Care management: agreement between nursing prescriptions and patients' care needs

**DOI:** 10.1590/1518-8345.0645.2723

**Published:** 2016-08-08

**Authors:** Marília Silveira Faeda, Márcia Galan Perroca

**Affiliations:** 1 RN, Hospital de Base de São José do Rio Preto, São José do Rio Preto, SP, Brazil. Master's Student, Faculdade de Medicina de São José do Rio Preto, São José do Rio Preto, SP, Brazil.; 2 PhD, Professor, Departamento de Enfermagem Especializada, Faculdade de Medicina de São José do Rio Preto, São José do Rio Preto, SP, Brazil.

**Keywords:** Patient Care Planning, Nursing Care, Nursing Process, Needs Assessment, Nursing

## Abstract

**Objectives:**

analyze agreement between nursing prescriptions recorded in medical files and patients' care needs; investigate the correlation between the nurses' professional background and agreement of prescriptions.

**Method:**

descriptive study with quantitative and documentary approach conducted in the medical clinic, surgical, and specialized units of a university hospital in the interior of São Paulo, Brazil. The new validated version of a Patient Classification Instrument was used and 380 nursing prescriptions written at the times of hospital admission and discharge were assessed.

**Results:**

75% of the nursing prescriptions items were compatible with the patients' care needs. Only low correlation between nursing prescription agreement and professional background was found.

**Conclusion:**

the nursing prescriptions did not fully meet the care needs of patients. The care context and work process should be analyzed to enable more effective prescriptions, while strategies to assess the care needs of patients are recommended.

## Introduction

Documentation is an essential practice in nursing, with clinical and legal importance, as well as being a relevant tool of communication among healthcare workers. Nurses are responsible for keeping precise and complete records to ensure the continuity, safety and quality of care delivery^1^.

A Nursing Prescription (NP), which is part of documentation, is an instrument to guide the actions of the staff in the delivery of care to patients^2^. Identifying the care needs of patients is essential in order for nurses to write NP and identify necessary interventions so that individualized quality care is provided^3^. Patient Classification Instruments (PCI) have been used to identify patients' nursing care needs. Thus, a PCI is a tool that enables nurses to plan, implement, and assess the care process^3^ ^-^ ^4^.

In practice, difficulties have been reported in regard to the implementation of the nursing process, such as the incomplete or incorrect use when compared to what is taught in nursing schools^5^, deficient knowledge on the part of professionals^6^, and an excessive number of nurses' activities, such as having to manage care, people, and material and physical resources^7^. Work overload limits the time nurses spend with their patients and potentially impedes more efficient assessment.

Prescriptions are performed electronically in some hospitals. Even though this type of recording represents an advancement in nursing care as it standardizes^8^ procedures and saves time^9^, its use has generated concerns. One such concern involves the system's copy and past resource, which allows the inclusion of non-valid data that do not transmit the patient's actual situation, which has the potential to affect critical thinking, decision-making and the quality of care^9^.

Unplanned care often results in missed care. Missed care is defined as any aspect of care that is required by patients but is omitted (either in part or in whole) or delayed. Studies report the areas in which care is neglected by the nursing staff^10^ ^-^ ^11^ and the reasons for such occurrences^11^.

The way in which prescriptions have been distributed among work shifts and the prescription-to-nurse proportion adopted by some health facilities has been cause for concern. Thus, the identification of failures in the prescription process is essential, as are strategies to promote efficacious care that meet the needs of patients^12^. This study's objective was to answer the following questions: *do nursing prescriptions meet the needs of patients? Do prescriptions, written at the time of hospital discharge, present greater agreement with the care needs of patients than those written at the time of admission? Are there variables related to patients, workers or the unit that influences agreement of these prescriptions?*

## Objectives

To identify agreement between nursing prescriptions recorded on patients' charts and patients' care needs, and investigate correlation between the nurses' professional background, patients' variables, and agreement of prescriptions.

## Method

This is a documentary and descriptive study with a quantitative approach. The study's setting was a private university hospital, with extra capacity, located in the interior of São Paulo, Brazil. Data were collected between September 2013 and January 2014 in the medical clinic, surgical and specialized units providing care to patients under the Unified Health System (SUS). These units were selected due to the number of prescriptions written daily by a single nurse, usually on the night shift.

The nursing staff in the facility under study provides care through SAE (Systematization of Nursing Care), which is recorded in electronic databases considering data collection, diagnosis, prescriptions, and nursing assessment. This process is based on a conceptual model to meet basic human needs and uses NANDA International taxonomy (NANDA I)^13^.

Due to a lack of information in the literature addressing this subject to support statistical calculation regarding the number of subjects, we established the analysis of ten prescriptions per nurse prescriber. A preliminary survey determined that 19 nurses allocated in the units under study prescribed nursing care to patients on a daily basis. Hence, the sample was composed of 190 randomly selected patients (drawn from a list of inpatients) within the data collection period, totaling 380 prescriptions (190x2).

The NP written at the times of admission and discharge were investigated based on the hypothesis that the longer the interaction between nurses and patients over the period of hospitalization, the more effective the assessment of the patients' care needs and, consequently, the better adjusted the care planning. The patients' demographic and clinical data were collected from their electronic files.

The study was conducted in three different stages:

1. *Assessment of patients' care needs:* a new validated version of a Patient Classification Instrument for adult and pediatric^3^ ^,^ ^14^ patients composed of nine care areas was used. The total score classifies patients into four categories of care: minimum (9-12 points), intermediary (13-18 points), semi-intensive (19-24 points) and intensive care (25-36 points). The assessment of the instrument's psychometric properties revealed a predictive capacity of 99.6%^14^, that is, the instrument accurately measures patients' care needs. Three clinical nurses allocated in the units under study applied the PCI at the time of admission and hospital discharge. These nurses were trained to apply the instrument and then assessed to verify whether they held the same understanding regarding the scale. A test application obtained 98% of agreement.

2. *Analysis of nursing prescriptions and whether they met identified needs:* the electronic prescriptions were read and transcribed on a spreadsheet containing the following items: care to be provided, frequency of actions, and notes of one of the researchers. The instrument's areas of care were listed, excluding Planning and Coordination of the Care Process, because it refers to the way care is systematized, to multidisciplinary participation, and resources - which is not the focus of this study.

Afterwards, using the cross-mapping method^15^, the prescriptions items were related to the eight PCI areas considering identical, synonymous, similar or related terms. This procedure was independently performed by two researches (with Master's and PhD degrees in nursing). In case of disagreement, a third researcher would be consulted; however, this was not necessary, because 100% of agreement was achieved. 

3. *Outline of the backgrounds of the nurses who wrote the prescriptions and characterization of the units under study:* a semi-structured questionnaire was applied containing information concerning demographic data (age, sex), workers (professional experience and background), and the unit's characteristics (number of collaborators and prescriptions per nurse).

The study was approved by the Institutional Review Board (Report No. 216.781/2013) and all the participants signed free and informed consent forms after receiving clarification regarding the study and its objectives.

Data were collected using Graph Pad Prism 5 (Graph Pad Software Inc., San Diego, CA, USA) and the following analyses were conducted:

- descriptive statistics to organize the data set, presented in frequencies, means, and standard deviations;
- Pearson's coefficient to verify the correlation between the nurses' backgrounds, patients' variables and care needs, and the variable agreement of prescriptions, considering the following: ≥0.6 (strong correlation); 0..3 ≤ and <0.6 (moderate correlation); <0.3 (weak correlation)^16^;
- The unpaired t-test was used to compare care included in the nursing prescriptions and the patients' care needs as identified using PCI. Level of significance was established at 0.05.

## Results

The study was composed of 380 nursing prescriptions (admission and discharge) concerning 190 patients. Most patients, 110 (58%), were women, and were 45 years old, on average (Standard deviation: sd=22 years) ranging from 1 to 93 years. They were hospitalized in the following specialties: Medical clinic 63 (33.1%), Infectious and Parasitic Diseases (IPD) 11 (5.8%), Pediatrics 16 (8.4%), Cardio/hematology 19 (10%), Gynecology and obstetrics (GO) 19 (10%), Neurology and orthopedics 23 (12.1%), General surgery 26 (13.8%), and Transplantation 13 (6.8%).

The classification of the patients' care needs, both at the time of admission and at discharge, revealed that minimum (45.3% and 49%) and intermediary care (31.6% and 30.2%) was more frequently prescribed. The length of hospitalization was 15.7 (sd=12) days on average, ranging from 1 to 130 days.

### Nurse prescribers

Of the 19 nurses participating in the study, 11 were women and eight were men, aged 32 years, on average (sd=7; variation from 24 and 49 years old); with an average professional experience of seven years (sd=4; variation of 3-15 years); having worked in the unit from seven months to four years. In regard to educational background, only four had graduated in nursing; three concluded an improvement program; ten attended a specialization program (with the most frequent specialties being Emergency and Urgent Care, Pediatrics and Neonatal, Nursing Management), and two held Master's degrees in nursing.

The nurses reported they wrote nursing prescriptions to an average of 35 patients daily, ranging from 10 (Palliative Care unit) to 73 patients (Emergency Department). Additionally, they were responsible for seven workers per duty, on average.

### Nursing prescriptions

The average number of care needs identified per patient ranged from 5.6 (IPD and GO) to 6.7 at time of admission and from 5.0 (medical clinics) to 8.9 (Neurology/orthopedics) at discharge (Table 1).

**Table 1:** Average number and absolute frequency of length of hospitalization (LH) and Care Needs (CN), according to the hospitalization unit (N=380). São José do Rio Preto, SP, Brazil, 2014

Units		LH (%)	CN (%)
Admission			
	Emergency	-	5.8 (9.3)
	Medical clinic	-	5.9 (9.5)
	IPD*	-	5.6 (9.0)
	Palliative Care	-	6.7 (10.8)
	Cardio/hematology	-	6.7 (10.8)
	Pediatrics	-	6.2 (10.0)
	GO^‡^	-	5.6 (9.0)
	Neurology/orthopedics	-	6.7 (10.8)
	Surgical clinic	-	6.2 (10.0)
	Transplantation	-	6.7 (10.8)
Discharge			
	Emergency	3.3 (1.9)	5.7 (9.5)
	Medical clinic	18.7 (11.0)	5.0 (8.5)
	IPD*	40.7 (23.9)	5.2 (8.7)
	Palliative Care	17.0 (10.0)	6.3 (10.5)
	Cardio/hematology	27.4 (16.1)	6.0 (10.0)
	Pediatrics	11.1 (6.6)	5.7 (9.5)
	GO^‡^	3.4 (2.0)	5.4 (9.0)
	Neurology/orthopedics	17.6 (10.3)	8.9 (14.9)
	Surgical clinics	4.7 (2.8)	5.6 (9.4)
	Transplantation	26.0 (15.4)	6.0 (10.0)

*IPD: Infectious and Parasitic Diseases; ‡GO: Gynecology and Obstetrics

The average number of nursing prescriptions ranged from 10.9 (5.4) at time of admission to GO to 19.7 (8.2) at the time of discharge from the Neurology/orthopedics unit. The average number of compatible items ranged from 6.9 (3.8) at the time of admission to the GO unit to 17.0 (8.1) at discharge from the Neurology/orthopedics. On average, 10.7 (4.0) compatible items were identified at time of admission and 11.6 (5.3) at discharge (Table 2).

**Table 2:** Comparison between the average number of nursing prescriptions and compatible items that met the patients' care needs per unit. São José do Rio Preto, SP, Brazil, 2014

Units		NP* M(sd)	CI† M(sd)
Admission			
	Emergency	14.3 (3.5)	9.2 (3.1)‡
	Medical clinic	13.2 (3.0)	7.5 (2.6)‡
	IPD^§^	13.3 (4.9)	8.3 (4.7)||
	Palliative Care	14.1 (3.0)	9.6 (3.1)‡
	Cardio/hematology	16.6 (4.7)	11.0 (3.6)‡
	Pediatrics	16.2 (4.2)	12.4 (4.2)‡
	GO**	10.9 (5.4)	6.9 (3.8)||
	Neurology/orthopedics	19.5 (7.0)	16.2 (6.8)††
	Surgical clinic	14.7 (3.9)	11.7 (3.6)||
	Transplantation	17.3 (4.7)	14.3 (4.8)††
	Total	15.0 (4.4)	10.7 (4.0)‡
Discharge			
	Emergency	15.5 (4.5)	10.1 (4.4)‡
	Medical clinic	16.0 (6.4)	11.1 (6.8)||
	DIP^§^	13.9 (5.2)	8.9 (5.4)††
	Palliative Care	14.9 (4.4)	10.4 (4.5)‡
	Cardio/hematology	18.5 (6.6)	13.0 (6.3)||
	Pediatrics	17.0 (5.2)	13.2 (5.1)||
	GO**	10.5 (5.1)	6.7 (3.6)‡
	Neurology/orthopedics	19.7 (8.2)	17.0 (8.1)††
	Surgical clinic	14.7 (4.0)	11.9 (3.8)||
	Transplantation	17.2 (5.2)	14.2 (5.2)††
	Total	15.8 (5.5)	11.6 (5.3)‡

*NP: Nursing prescriptions; †CI: Compatible items; ‡p<0.01; §IPD: Infectious and Parasitic Diseases; ||p<0.05; **GO: Gynecology and Obstetrics; ††NS (Not Significant)

Correlation between agreement of nursing prescriptions and the demographic profile of patients, length of hospitalization, and PCI score is presented in Table 3. Values between 0.02 and 0.88 (NP and PCI score) were found. 

**Table 3:** Agreement between nursing prescriptions, patient demographic data, length of hospitalization, and PCI average score per hospitalization unit. São José do Rio Preto, SP, Brazil, 2014

Units	Sex	Age	LH*	PCI score^†^
Emergency	0.15	0.10	0.29	0.19
Medical clinic	0.11	0.18	0.29	-0.16
IPD^‡^	0.46	0.38	0.45	0.08
Palliative Care	0.22	0.16	0.19	0.23
Cardio/hematology	0.19	-0.39	0.02	-0.17
Pediatrics	0.58	-0.16	0.24	0.62
GO^||^	0.00	0.71	0.46	0.88
Neurology/orthopedics	0.13	0.43	0.03	0.34
Surgical clinic	0.23	-0.05	0.23	-0.22
Transplantation	0.26	-0.07	0.19	0.02
Total	0.23	0.13	0.24	0.18

*LH: Length of hospitalization; †PCI: Patient Classification Instrument; ‡IPD: Infectious and Parasitic Diseases; ||GO: Gynecology and Obstetrics.

Correlation between agreement of nursing prescriptions, professional background, number of workers, and number of NP per nurse, indicated values that ranged from -0.02 (age and years of professional experience) to -0.53 (years working at the hospitalization unit and nursing prescriptions per nurse) (Table 4).

**Table 4:** Correlation between agreement of nursing prescriptions and nurse prescribers variables per hospitalization unit. São José do Rio Preto, SP, Brazil, 2014

Units	Sex	Age	TPE*	TPE/HI^†^	NC^‡^	NP/N§
Emergency	0.38	0.27	-0.38	-0.53	-0.51	-0.53
Medical clinic	0.13	-0.03	0.02	0.01	0.04	0.16
DIP^||^	0.23	0.36	0.38	0.21	-0.19	-0.23
Palliative care	0.00	0.02	0.09	0.00	-0.18	-0.17
Cardio/hematology	0.00	-0.04	-0.07	-0.08	-0.09	-0.08
Pediatrics	0.00	0.09	0.09	0.09	0.00	0.09
GO**	0.00	0.09	0.07	0.18	0.02	0.07
Neurology/orthopedics	-0.07	0.29	0.29	0.29	0.10	0.27
Surgical clinic	0.00	0.22	-0.14	-0.14	-0.26	-0.22
Transplantation	0.00	0.36	0.41	-0.43	0.41	-0.02
Total	0.06	0.16	0.07	-0.04	-0.06	-0.06

*TPE: Time of professional experience; †TPE/HU: Time of professional experience in the Hospitalization Unit; ‡NC: Number of Collaborators; §NP/N: Nursing Prescriptions per Nurse; ||IPD: Infectious and Parasitic Diseases; **GO: Gynecology and Obstetrics.

Considering the total number of items prescribed (3,120), we verified that 2,340 (75%) items met the identified patients' care needs. A total of 621 care needs were identified at the time of admission and 598 at the time of discharge. When compared with the items prescribed, we verified that 35% (admission) and 32.3% (discharge) had no correspondence with the prescriptions.

Care areas such as investigation and monitoring, mobility and activity and therapeutics required nursing care and were frequently included in the NP. Even though areas regarding body care and elimination and emotional support demanded attention, they were less frequently prescribed at both time of admission and discharge. Some care practices that were already integrated into the units' routines were prescribed, such as hand hygiene and keeping identification tags on the patients' left upper limbs. Correlation between agreement of NP and PCI areas of care are presented in Table 5. Both at time of admission and at discharge, the areas more frequently associated were investigation/monitoring (0.45 and 0.49) and therapeutics (0.42 and 0.47).

**Table 5:** Correlation between PCI care areas and agreement of nursing prescriptions. São José do Rio Preto, SP, Brazil, 2014

Units		A1*	A2^†^	A3^‡^	A4^§^	A5^||^	A6^¶^	A7**	A8^††^
Admission									
	Emergency	0.65	-0.03	0.18	0.12	0.29	0.18	-0.14	-0.23
	Medical clinic	0.38	0.02	0.04	0.07	0.34	0.33	-0.22	-0.02
	IPD^‡‡^	0.72	-0.07	0.09	0.01	0.19	0.94	0.27	0.04
	Palliative care	0.29	-0.17	0.07	-0.07	0.53	0.35	0.12	0.00
	Cardio/hematology	0.53	0.09	0.11	-0.03	0.72	0.21	0.00	0.09
	Pediatrics	0.72	0.13	-0.02	0.00	0.41	0.16	-0.02	0.00
	GO^||||^	0.26	-0.14	0.00	0.00	0.43	0.54	-0.04	0.03
	Neurology/orthopedics	0.09	0.01	0.00	0.00	0.17	0.46	0.00	0.13
	Surgical clinic	0.36	-0.38	0.03	0.04	0.18	0.53	-0.23	0.21
	Transplantation	0.53	-0.02	0.14	0.08	0.23	0.52	-0.26	-0.06
	Total	0.45	-0.05	0.06	0.02	0.34	0.42	-0.05	0.01
Discharge									
	Emergency	0.46	-0.02	0.23	0.10	0.34	0.22	-0.14	-0.23
	Medical clinic	0.52	0.07	0.00	0.09	0.71	0.31	-0.17	-0.03
	IPD^‡‡^	0.81	-0.03	0.13	-0.03	0.52	0.97	0.24	0.01
	Palliative care	0.73	-0.16	0.09	-0.04	0.53	0.44	0.19	0.00
	Cardio/hematology	0.53	0.17	0.16	0.01	0.41	0.36	0.03	0.07
	Pediatrics	0.62	0.21	0.00	0.00	0.24	0.20	0.01	0.00
	GO^||||^	0.46	-0.17	0.04	0.02	0.45	0.51	-0.02	0.11
	Neurology/orthopedics	0.34	0.06	0.00	0.00	0.17	0.42	0.00	0.18
	Surgical clinic	0.23	-0.38	0.05	0.02	0.23	0.66	-0.20	0.21
	Transplantation	0.19	0.07	0.24	0.09	0.34	0.62	-0.33	-0.05
	Total	0.49	-0.01	0.09	0.02	0.40	0.47	-0.03	0.02

A1*: Investigation/Monitoring; A2†:Body care/Eliminations; A3‡: Skin/mucosa care; A4§: Nutrition/Hydration; A5||: Mobility/Activity; A6¶: Therapeutics; A7**: Emotional support; A8††: Health Education; ‡‡IPD: Infectious and Parasitic Diseases;; ||||GO: Gynecology and Obstetrics.

## Discussion

Patient-centered care, that is, individualized care that ensures the participation of patients in decision-making regarding their health/disease situation, is considered to be vital in nursing practice^17^ **_._**

To provide a perspective focused on the patients' care needs, the Brazilian Council of Nursing^18^, in 2009, began regulating the Systematization of Nursing Care in healthcare facilities. Its methodological instrument, the Nursing Process, guides care and the documentation of professional practice in its various stages^19^.

The operationalization of this method of organizing care represents an important challenge, as shown by this study's findings. We verified that only 75% of the NP items were compatible with the patients' care needs, indicating that the nurses' prescriptions do not fully meet the patients' needs. Additionally, 35% of the needs identified at the time of admission and 32.3% of those identified at hospital discharge did not correspond with the prescriptions.

These findings lead us to reflect on how prescriptions have been used in hospital facilities. Undoubtedly, its incorporation into the professional routine meant significant improvement in terms of providing quality, individualized, safe, and result-focused care to patients^20^; however, there is still a need to investigate the context of care and work process in which such a procedure is implemented to make necessary adjustments.

Difficulties implementing the nursing process, and consequently prescriptions, have been indicated^5^ ^,^ ^7^ ^,^ ^9^. Some of these difficulties may have influenced this study's results.

Many Brazilian hospital facilities have used electronic nursing documentation and, when properly performed, this favors a practice that enables the frequent review of care plans and the implementation of changes whenever necessary^21^. We noticed in the facility under study, however, that prescriptions from the previous day were copied and care needs were not fully considered or not updated, while prescriptions included procedures that had already been implemented in the units' routines.

The system's resources, such as the ability to copy and paste, favors easy reproducibility of prescriptions so that nurses often fail to use clinical rationale to reassess their patients' care needs. This omission of care influences decision-making and may interfere in the quality of care delivery^9^. Accessing precise information concerning a patient's health status using electronic nursing documentation is highly relevant^22^, but its inappropriate use has been reported by researchers^9^ ^,^ ^23^.

Another factor that may interfere in NP's agreement with patients' care needs is workload. Nurses from the night shift are usually the ones responsible for prescriptions. Those working on this shift assume responsibility for all the patients in the unit (an average of 35) and also monitor the activities of their collaborators, in addition to admitting patients, which requires a considerable amount of time. Work overload decreases the time nurses have available to establish bonds with patients and determine those needs requiring interventions^24^. Limited time to properly develop NP was also verified in another health care setting^25^.

The analysis of the nursing prescriptions' content revealed that nurses prescribe a larger number of care actions related to the control of vital signs, airway maintenance and compliance with measurement scales, in addition to mobility and therapeutics. Body care, however, such as oral hygiene and elimination care, were the most frequently neglected.

The occurrence of so-called missed care has been reported in studies^10^ ^-^ ^11^. Nine basic care elements were reported as being regularly omitted, such as: walking, nutrition, patient education, preparation for discharge, emotional support, hygiene, intake/elimination documentation, and surveillance^10^. The factors that predict such omissions include the shift worked, number and allocation of personnel, communication within the multidisciplinary team, intensity of workload, and satisfaction of nurses with their current job^11^.

It is believed that the longer nurses monitor a patient, the more efficiently they identify the patient's needs. No statistical significance, however, was found between the agreement of prescriptions written at the time of admission and at discharge, even though patients were discharged on the 17^th^ day of hospitalization, on average. This reinforces the need to rethink this facility's care organization model.

In addition to attention being paid to factors linked to environment and the way work is performed, the use of assessment strategies can contribute to more effective care planning, minimizing missed care. We recommend the adoption of instruments to make such assessments in order to guide critical thinking when identifying patients' nursing care needs^14^ ^,^ ^26^. The use of scales to conduct assessments has recently been verified to enable the identification of a larger number of care areas^27^.

It is not yet possible to establish whether nursing care needs that go unsatisfied could be used as an indicator of the quality of nursing care provided in hospitals or what is the best method to ensure that care plans and nursing prescriptions meet all of a patient's care needs^28^.

Even though this study's results have showed weak correlation between the number of NP/nurse and agreement, we believe it is infeasible for a nurse to prescribe for such a large number of patients. The literature does not report the proportion of prescriptions recommended per nurse such that a deeper discussion could be grounded. The indication that nurses' prescriptions do not fully meet the care needs of patients, however, shows that this work organization may not be efficacious and demands managers to take a closer look.

A limiting aspect of this investigation is the fact it was restricted to one hospital facility and did not include all its hospitalization units. Thus, replication of the study is necessary to verify how prescriptions are developed in other settings. 

## Conclusion

This investigation revealed that nursing prescriptions did not fully meet patients' care needs. Additionally, neither do the time the prescription is written nor the professionals' or the units' characteristics impact level of agreement. Monitoring and analysis of the context in which care is provided and work processes occur is recommended to enable more effective prescriptions, in addition to the use of strategies to assess patients' care needs.
